# Control and complications of diabetes in urban primary care units in Thailand: a cross-sectional study

**DOI:** 10.1186/s12875-022-01823-7

**Published:** 2022-08-22

**Authors:** Thanapat Puangpet, Tanyaporn Pongkunakorn, Nahathai Chulkarat, Chutikan Bunlangjit, Apinya Surawit, Bonggochpass Pinsawas, Pichanun Mongkolsucharitkul, Korapat Mayurasakorn

**Affiliations:** 1 Department of Social Medicine, Samutsakhon Hospital, Samutsakhon, Thailand; 2 Population Health and Nutrition Research Group, Siriraj Medical Research Center, Faculty of Medicine Siriraj Hospital, Mahidol University, Bangkok, Thailand

**Keywords:** Diabetes registry, Glycemic control, Quality of care, Primary care units

## Abstract

**Background:**

Primary health care system plays a central role in caring for persons with diabetes. Thai National Health Examination Survey (NHES) reports that only 40% of patients with type 2 diabetes mellitus (T2DM) achieve optimal glycemic control. We sought to evaluate the quality of diabetic care (QOC), prevalence of microvascular complications, and associated risk factors among T2DM patients treated at primary care units in urban areas in Thailand.

**Methods:**

A population-based, cross-sectional study of 488 T2DM patients aged over 35 years from 25 primary care units in Samutsakhon, Thailand was conducted during February 2018 to March 2019. Clinical targets of care (TOC) and processes of care (POC) were measured to evaluate QOC. Multivariate logistic regression models were applied to explore the association between risk factors and glycemic control.

**Results:**

41.2% of women and 44.4% of men achieved hemoglobin A1C (A1C) < 53 mmol/mol, while 31.3% of women and 29.7% of men had poor glycemic control (A1C > 63 mmol/mol). 39 participants (8%) achieved all TOC and 318 participants (65.2%) achieved all POC. Significant risk factors for poor glycemic control included diabetes duration > 6 years (AOR = 1.83, 95% CI = 1.20–2.79), being overweight (AOR = 2.54, 95% CI = 1.58–4.08), obesity (AOR = 1.71, 95% CI = 1.05–2.89), triglycerides > 1.7 mmol/l (AOR = 1.81, 95% CI = 1.25–2.78), low density lipoprotein-cholesterol (LDL-C) ≥ 2.6 mmol/l (AOR = 1.55, 95% CI = 1.04–2.28). On the other hand, participants aged > 65 years (AOR = 0.25, 95% CI = 0.14–0.55) or achieved TOC indicators (AOR = 0.69, 95% CI = 0.43–0.89) were significantly associated with glycemic control. Diabetic retinopathy was significantly related to obesity (AOR = 2.21, 95% CI = 1.00–4.86), over waist circumference (AOR = 2.23, 95% CI = 0.77–2.31), and diastolic blood pressure > 90 mmHg (AOR = 1.81, 95% CI = 1.48–1.96).

**Conclusion:**

Access to essential diabetic screening in primary care units is crucial to determine status of disease control and guide disease management. Duration of T2DM, high body mass index, triglyceride and LDL-C were independently associated with poor glycemic control. Obesity was highly associated with diabetes retinopathy. Effort should be taken seriously toward monitoring these factors and providing effective care.

## Introduction

Type 2 diabetic mellitus (T2DM) is one of increasingly prevalent non-communicable diseases (NCDs) worldwide. According to Thai National Health Examination Surveys (NHES) during 2004–2014 [1–3] and the International Diabetes Federation [4], the prevalence of Thai T2DM increased from 7.7% in 2004 to 7.8% in 2009, then to 9.9% in 2014. Diabetes is the leading cause of chronic kidney disease and death worldwide [5]. Thanks to a national program in Thailand to promote access to assessment of people at an increased risk for NCDs, the proportion of Thai persons with undiagnosed diabetes declined from 65.2% to 51.2% in men and from 48.5% to 41.3% in women between 2004 and 2014 [1–3]. The proportion of well-controlled glycemia improved from 15% in 2004 to 36% in 2012, and to 40% in 2014 [6]. Nearly half of the direct medical cost of diabetes is allocated to hospital care or the cost of managing complications, and only 14% to outpatient or community care [7].

Diabetic persons with complications require 2.3–18.5 times more resources, and suffer poorer quality of life [7]. Growing inequalities in healthcare for people who live in rural and urban communities are observed generally [8]. Recent data in Thailand have shown that individuals with T2DM treated at regional hospitals had a better glycemic control than that of individuals from local hospitals. This implies that more comprehensive care provided by a medical team in regional hospitals and more urbanized patients may produce better clinical outcomes or high rates of awareness of diabetes than in rural patients [9]. In addition, education and behavioral support are especially crucial for individuals attempting for improvement of T2DM and prevention of early complications. Since 2002, concerted efforts have been made to institute comprehensive care to most diabetic individuals through the primary health care system, and particularly to those living in communities under the universal health care scheme (UHC). This provides free health care at the point of service in order to guarantee access to essential health care at primary care units (PCUs) [10]. Moreover, a resilient health system and management support team are a fundamental tenet of the diabetes care model linked to improved outcomes of diabetes control [11]. Services include health promotion, education, and screening for risk factors that lead to diabetes complications driven by interdisciplinary collaboration, proactive policies, and holistic care approaches [10, 12, 13]. This concept is also aligned with the guidelines of team-based care outlined by the Institute of Medicine (IOM) and the American Association of Colleges of Nursing (AACN), which recognize the patient as the center of the care team and must be aligned with cost containment mechanisms of a UHC to ensure long-term governmental financial sustainability [11].

To improve diabetes outcomes and actively prevent complications, it is useful to assess and monitor the quality of care (QOC), access to processes of care (POC) and achievement of clinical targets of care (TOC) [14, 15]. These metrics are associated with reduction and delayed onset of microvascular and macrovascular complications [15–19]. For instance, maintenance of blood pressure below 140/90 mmHg is recognized to reduce cardiovascular events and microvascular complications such as stroke, albuminuria, retinopathy, and chronic kidney disease (CKD), leading to overall benefits for populations at risk [14, 20–23]. Subgroup analysis of diabetic participants and large meta-analyses demonstrated a significant reduction in all-cause deaths for every 1.01 mmol/l reduction in low density lipoprotein-cholesterol (LDL-C) [24] and the protective benefits of statin, thereby reducing fatal and non-fatal cardiovascular outcomes [25, 26]. Diabetic nephropathy (DN) and diabetic retinopathy (DR) are clearly associated with poor glycosylated A1C and prolonged duration of diabetes [15, 19]. Optimal glycemic and blood pressure control can attenuate the progression of CKD and vision-threatening DR [27]. Interestingly, the most common barriers to glycemic control include improper psychological/support, low socioeconomic status, and insufficient access to essential care [28].

Therefore, we aimed to evaluate the results of T2DM care and determine the association between quality of glycemic control and the care system through accessibility of diabetic complication screening, and successful achievement of outcome goals in urban areas of Samutsakhon province, Thailand.

## Materials and methods

### Study population

Baseline data were collected from 25 primary care units in 18 urban communities, in Samutsakhon province, Thailand. Among 13,256 patients, 6,871 had been diagnosed with T2DM whose care was provided by collaborative primary care providers (PCP) including family physicians, general practitioners, pharmacists and nurses, and the average wait time to visit a PCP was 2–3 months. Approximately 97% of these patients were all-insured, indicating there was no significant discrepancy in quality of diabetes care. Complication screening and complete blood measurement were set up during the annual checkup campaign of each PCU. All primary care units operated under a standard protocol that included guidelines of diabetic treatments according to the American Diabetes Association (ADA)’s recommendations [29] and the Thai clinical practice guideline for diabetes [30], standard anti-diabetic drugs, clinical investigations, and referral system. Participants were recruited in routinely scheduled visits during February 2018 to March 2019. Written informed consent was obtained from all participants. All procedures were approved by the Siriraj Institutional Review Board (095/2560 (EC1)). Participants were eligible for inclusion if they had received care and treatment for T2DM at least for 12 months. Pregnant participants and those with gestational diabetes or at their postpartum stage were excluded. Data were collected through face-to-face interviews and a case record form was completed by research coordinators using standardized questionnaires.

### Data collection

Standardized questionnaires comprised demographic data and self-reported comorbidities such as hypertension, dyslipidemia, gout, chronic kidney disease, and a family history of NCDs. Anthropometric information was retrieved from an electronic medical record. Date of diagnosis, behavioral risk factors, and lifestyle (physical activities, smoking and alcohol consumption) were also collected. The recent use of antidiabetic agents, antihypertensive agents, lipid lowering agents and antiplatelet therapies were retrieved from the medical record. Physical activities or exercise were classified into three categories: (1) less than once a week or seldom, (2) once a week, (3) at least 2–3 times a week. Smoking was classified into three categories: (1) never, (2) quit smoking, and (3) current smoking. Alcohol consumption was classified into two categories: (1) yes or drinking and (2) no or never drinking. Body mass index (BMI) was classified as (1) underweight < 18.5 kg/m^2^, (2) normal 18.5–22.9 kg/m^2^, (3) overweight 23.0–24.9 kg/m^2^, (4) obese I 25.0- 29.9 kg/m^2^, and (5) obese II ≥ 30.0 kg/m^2^. Waist circumference (WC) was classified as (1) normal (< 90 cm in men and < 80 cm in women) and (2) over. Optimal blood pressure (BP) was defined by systolic blood pressure (SBP) < 140 mmHg and diastolic blood pressure (DBP) < 90 mmHg as defined the American College of Cardiology/American Heart Association Task Force and the American Diabetes Association [14, 31].

### Measurements

Laboratory data including fasting blood glucose (FBG), A1C, total cholesterol (TC), triglycerides (TG), high-density lipoprotein (HDL-C) were retrieved from the medical record within the preceding 9 months. LDL-C was estimated based on the Friedewald formula [32]. Plasma uric acid levels, liver functions (aspartate transaminase; AST, and alanine aminotransferase; ALT) and electrolytes (sodium; Na^+^ and potassium; K^+^) were determined by a Cobas 6000 clinical chemistry analyzer (Roche Diagnostics, Basel, Switzerland).

### Quality of Care (QOC) assessment

QOC [6, 15] comprised TOC and POC. TOC included the *ABC* indicators of diabetes: *A*-A1C, *B*-BP, and *C*-LDL-C. POC was the receipt of essential diabetes examinations, including the *FACE* indicators of diabetes: *F*-Foot exam, *A*-A1C exam, *C*-LDL-C exam, and *E*-eye exam. In addition, aggregates of the QOC measures were generated for TOC (*AllABC)*, POC (*AllFACE)*, and all QOC indicators (a combination of TOC and POC). *AllABC* represented all three of the treatment goals achieved. *AllFACE* represented an indicator variable for cases where all four POC were conducted. *All7Q* represented an indicator variable of whether all seven of the clinical indicators were achieved, including *AllABC* and *AllFACE*. Finally, the count variable *Num7Q* represented the number of TOC and POC outcomes achieved *(0, 1, 2, 3, 4, 5, 6 or 7).* Participants were considered as satisfactory for achievement of clinical targets if *A*-A1C ≤ 53 mmol/mol, *B*-BP ≤ 130/80 mmHg, and *C*-LDL-C < 2.6 mmol/l. Participants were considered to have satisfactorily met the examination target if they were examined once in the previous 12 months for A1C, cholesterol, feet and eyes. Moreover, all QOC was also compared the effectiveness with previous studies.

### Complication data collection

Data were collected on complications including cerebrovascular accident, cerebral infarction, ischemic stroke, hemorrhagic stroke, DR, DN, renal insufficiency, and neuropathy. Retinal photography during the year prior to enrollment was retrieved from the medical record. If this had not been performed, subjects were scheduled for retinal screening using a non-mydriatic seven-field stereoscopic retinal photography (KOWA nonmyd 8S, Kowa, Tokyo, Japan) by a trained technical specialist. Results were verified by an ophthalmologist. DR was defined as the presence of at least one microaneurysm, hemorrhage, or evidence of proliferative retinopathy. DR was reported into three categories: (1) normal or no-DR, (2) non-proliferative diabetic retinopathy (NPDR; mild, moderate or severe), and (3) proliferative diabetic retinopathy (PDR). Techniques and standards [33, 34] for diagnosing retinopathy did not change over the period of the study. To assess renal insufficiency, spot morning urine collected at the clinics was sent to the main lab facility, and stored at 4 °C until processing. To determine albuminuria, spot morning urine albumin was measured by an immunoturbimetric assay and creatinine using the enzymatic colorimetric method. Then, the urine albumin-to-creatinine ratio (ACR) was calculated. Staging of DN was categorized according to the Kidney Disease Improving Global Outcomes (KDIGO) 2012 guidelines [35, 36]: microalbuminuria and macroalbuminuria as well as serum creatinine (Cr), estimated glomerular filtration rate (eGFR). The presence of neurological problems of the feet was evaluated using monofilament (10 ng). Skin and nail characteristics, the presence of foot deformities, and the risk of foot ulcers were evaluated by well-trained nurses in all participants. Briefly, nurses inspected for visible abnormalities, peripheral pulse palpation and monofilament testing [37].

### Statistical analyses

Continuous variables were described using means, and standard error of mean (SEM), whereas categorical variables were summarized using counts and percentages. Comparisons between groups were analyzed by independent t-test or Chi-square test, when appropriate. Logistic regression was used to estimate odds ratio (OR) and 95% confidence interval (95% CI) for predictive factors associated with glycemic control. All risk factors were analyzed first in univariate logistic analyses and included in the initial multivariate model if an association existed (*p*-value < 0.2). Variables were eliminated in the final model, if they were not associated with glycemic control (*p*-value < 0.05). Confounders were identified if they altered the remaining coefficients by greater than 20% after their removal from the model. If a variable was identified as a confounder, it was forced into the final model. Our results indicate that age and HDL were confounding factors of glycemic control. Therefore, we have addressed the confounding variables appropriately. Statistical analyses were performed using STATA version 15.0 (Stata Corporation, College Station, TX, USA).

## Results

### General characteristics of the study participants

Four hundred and eighty-eighth participants were enrolled with the average age of 64.3 ± 0.61 years in women and 63.4 ± 1.07 years in men (Table 1). The average duration of diabetes in women was greater than in men (7.4 ± 0.28 years vs. 5.4 ± 0.34 years, *p* = 0.002). Based on BMI and WC, approximately two-third of participants were obese. No differences between genders were observed in SBP and self-reported underlying diseases. Men were more likely to consume alcohol and to be current smokers than women (*p* < 0.001).

**Table 1:** General characteristics of diabetic participants

Variables	Men	Women	*p*-value*
Number, (%)	125 (25.61)	363 (74.39)	
Age, years	63.4 ± 1.07	64.3 ± 0.61	0.442
< 40, n (%)	4 (3.20)	9 (2.49)	0.259
40—59, n (%)	45 (36.00)	98 (27.07)	
60—79, n (%)	66 (52.80)	224 (61.88)	
> 80, n (%)	10 (8.00)	31 (8.56)	
Duration of disease, years	5.4 ± 0.34	7.4 ± 0.28	0.002
< 5, n (%)	58 (47.54)	93 (25.98)	< 0.001
5—9, n (%)	53 (43.44)	217 (60.61)	
10—14, n (%)	7 (5.74)	27 (7.54)	
> 15, n (%)	4 (3.28)	21 (5.87)	
BMI, kg/m^2^	26.5 ± 0.49	27.1 ± 0.27	0.290
< 18.5	7 (5.60)	12 (3.31)	0.208
18.5—22.9	30 (24.00)	59 (16.25)	
23.0—24.9	18 (14.40)	65 (17.91)	
25.0—29.9	44 (35.20)	135 (37.19)	
> 30.0	26 (20.80)	92 (25.34)	
NC, cm	35.4 ± 0.78	34.0 ± 0.53	0.167
WC, cm	91.0 ± 1.29	89.1 ± 0.71	0.179
m ≤ 90 cm, w ≤ 80 cm	54 (43.55)	83 (23.12)	< 0.001
m > 90 cm, w > 80 cm	70 (56.45)	276 (76.88)	
SBP, mmHg	133.8 ± 1.36	133.1 ± 0.78	0.664
< 130, n (%)	44 (35.20)	147 (40.50)	0.656
130—139, n (%)	44 (35.20)	108 (29.75)	
140—159, n (%)	32 (25.60)	94 (25.90)	
> 160, n (%)	5 (4.00)	14 (3.86)	
DBP, mmHg	76.0 ± 1.07	72.9 ± 0.60	0.010
**Underlying diseases**			
Hypertension, n (%)	102 (81.60)	288 (79.34)	0.586
Dyslipidemia, n (%)	73 (58.40)	221 (60.88)	0.635
Ischemic heart, n (%)	3 (2.40)	5 (1.38)	0.437
Stroke or paralysis, n (%)	2 (1.60)	5 (1.38)	0.572
Gout, n (%)	14 (11.20)	31 (8.54)	0.374
**Lifestyle**			
Exercise, n (%)			
seldom	59 (47.58)	171 (47.50)	0.988
once a week	25 (20.16)	113 (31.39)	
> 2–3 times a week	40 (32.26)	76 (21.11)	
Smoking, n (%)			
never	78 (63.41)	354 (98.06)	< 0.001
stop smoking	36 (29.27)	6 (1.66)	
smoking	9 (7.32)	1 (0.28)	
Alcohol consumption, n (%)			
no	84 (67.74)	350 (96.69)	< 0.001
yes	40 (32.26)	12 (3.31)	
**Family history of CVD**			
Heart failure, n (%)	2 (1.60)	26 (7.16)	0.021
Cancer, n (%)	20 (16.00)	67 (18.46)	0.536
Ischemic heart, n (%)	1 (0.80)	3 (0.83)	0.728
Paralysis or stroke, n (%)	5 (4.00)	17 (4.68)	0.488

^*^p-value is the differences between male and female based on independent t-test or chi-square for continuous and categorical variables, respectively; mean ± SEM, standard error of mean, *BMI* Body mass index, *NC* Neck circumference, *WC* Waist circumference, *SBP* Systolic blood pressure, *DBP* Diastolic blood pressure, *CVD* cardiovascular diseases

### Clinical characteristics

41.2% of women and 44.4% of men achieved optimal glycemic control as defined by A1C < 53 mmol/mol. One third of both genders had poor glycemic control (A1C > 63 mmol/mol). Women had significantly higher HDL-C levels than men (1.32 ± 0.17 vs. 1.23 ± 0.03 mmol/l, *p* = 0.009). 279 participants (61.4% in men and 60.2% in women) had an LDL-C level of < 2.6 mmol/l and 354 participants (71.4% in men and 76.2% in women) had an eGFR > 59 ml/min/1.73m^2^ (Table 2).

**Table 2:** Biochemical data of diabetic participants

Variables	Men	Women	*p*-value*
FBG, mmol/l	8.32 ± 0.20	8.26 ± 0.146	0.831
< 5.5, n (%)	5 (4.13)	41 (11.36)	0.047
5.5—6.9, n (%)	36 (29.75)	87 (24.10)	
> 6.9, n (%)	80 (66.12)	233 (64.54)	
A1C, mmol/mol	58.38 ± 2.09	59.84 ± 1.07	0.515
< 53, n (%)	48 (44.44)	142 (41.16)	0.912
53—63.9, n (%)	28 (25.93)	95 (27.54)	
64—74.9, n (%)	14 (12.96)	41 (11.88)	
75—107.9, n (%)	16 (14.81)	56 (16.23)	
> 107.9, n (%)	2 (1.85)	11 (3.19)	
TC, mmol/l	4.38 ± 0.11	4.51 ± 0.54	0.234
< 5.2, n (%)	89 (76.07)	265 (75.93)	0.776
5.2—6.2, n (%)	18 (15.38)	60 (17.19)	
> 6.2, n (%)	10 (8.55)	24 (6.88)	
TG, mmol/l	1.57 ± 0.8	1.61 ± 0.39	0.611
< 1.7, n (%)	80 (68.97)	235 (67.14)	0.716
1.7—5.6, n (%)	36 (31.03)	115 (32.86)	
> 5.6, n (%)	0 (0.00)	0 (0.00)	
HDL-C, mmol/l	1.23 ± 0.03	1.32 ± 0.17	0.009
m < 1, w < 1.3, n (%)	32 (27.83)	169 (48.29)	< 0.001
m ≥ 1, w ≥ 1.3, n (%)	83 (72.17)	181 (51.71)	
LDL-C, mmol/l	2.47 ± 0.09	2.47 ± 0.48	0.945
< 2.6, n (%)	70 (61.40)	209 (60.23)	0.181
2.6—3.3, n (%)	26 (22.81)	83 (23.92)	
3.4—4.1, n (%)	8 (7.02)	40 (11.53)	
> 4.1, n (%)	10 (8.77)	15 (4.32)	
Cr, mmol/l	13.65 ± 1.49	9.23 ± 0.33	< 0.001
eGFR, ml/min/1.73m^2^	77.0 ± 2.12	79.7 ± 1.74	0.399
≥ 90 (G1), n (%)	39 (32.77)	140 (39.66)	0.700
60—89 (G2), n (%)	46 (38.66)	129 (36.54)	
45—59 (G3A), n (%)	21 (17.65)	49 (13.88)	
30—44 (G3B), n (%)	12 (10.080	29 (8.22)	
15—29 (G4), n (%)	1 (0.84)	5 (1.42)	
< 15 (G5), n (%)	0 (0.00)	1 (0.28)	
AST (SGOT), u/l	29.0 ± 2.87	30.2 ± 6.26	0.893
ALT (SGPT), u/l	32.2 ± 5.60	24.7 ± 4.43	0.329
Uric acid, mmol/l	0.40 ± 0.05	0.32 ± 0.01	0.023
Blood Na^+^, mmol/l	135.0 ± 5.00	180.0 ± 46.04	0.611
Blood K^+^, mmol/l	4.1 ± 0.34	3.7 ± 0.03	0.085

^*^p-value is the differences between male and female based on independent t-test or chi-square for continuous and categorical variables, respectively; mean ± SEM; *FBG* Fasting blood glucose, *A1C* Hemoglobin A1c, *TC* Total cholesterol, *TG* Triglyceride, *HDL-C* High-density lipoprotein, *LDL-C* Low-density lipoprotein, *eGFR* Estimated glomerular filtration rate, *Cr* Serum creatinine, *AST* Aspartate transaminase, *SGOT* Serum glutamic oxaloacetic transaminase, *ALT* Alanine aminotransferase, *SGPT* Serum glutamate-pyruvate transaminase, *Na*^+^ Sodium electrolyte, *K*^+^ Potassium electrolyte, *SEM* Standard error of mean

### QOC

Nearly 40% of participants achieved glycemic and blood pressure targets (Table 3). Only 39 participants (8.0%) achieved *AllABC* goals and 28 participants (5.7%) achieved *all7Q* goals, while 318 participants (65.2%) achieved *AllFACE*. DR and risk of foot ulcers were found in 47 (13.6%) and 40 (8.9%) participants, respectively (Table 4). Albuminuria was tested in only 12 participants (data not shown); therefore,the prevalence of DN was not estimated.

**Table 3:** Quality of diabetic care among T2DM at primary care units

**Quality of care indicators**		Case n	%
**TOC**	A; A1C < 53 mmol/mol	190	41.9
	B; BP < 130/80 mmHg	191	39.1
	C; LDL-C < 2.6 mmol/l	279	60.5
*AllABC* goals		39	8.0
**POC**	F; Foot examination	453	92.8
	A; A1C examination	453	92.8
	C; LDL-C examination	466	95.5
	E; Eye examination	356	73.0
*AllFACE* goals		318	65.2
**Number of 7Q**	1	7	1.4
	2	16	3.3
	3	30	6.2
	4	108	22.1
	5	162	33.2
	6	137	28.1
*All7Q*		28	5.7

*A1C* Hemoglobin A1c, *BP* Blood pressure, *LDL-C* Low-density lipoprotein, *TOC* Clinical target of care, *POC* Process of care

**Table 4:** The prevalence of an each stage in diabetic complications

**Diabetic complications**	Case n	%
Diabetic retinopathy		
no DR	297	86.3
NPDR; mild	39	11.3
NPDR; moderate	8	2.3
NPDR; severe	0	0.0
PDR	0	0.0
Risk of foot ulcers		
mild risk	413	91.2
moderate risk	18	4.0
severe risk	22	4.9

*DR* Diabetic retinopathy, *NPDR* Non-proliferative diabetic retinopathy, *PDR* Proliferative diabetic retinopathy

### Logistic regression models

The association between variables and glycemic control was analyzed by multivariate logistic regression (Table 5). Risk factors independently related to the quality of glycemic control (referred by A1C < 53.0 vs. ≥ 53.0 mmol/mol) including age, overweight and obesity, diabetes duration, TG, LDL-C, and TOC achievement. Older patients (age > 65 years) achieved better glycemic control than younger patients. Glycemic control was negatively associated with being overweight (AOR = 2.54, 95% CI = 1.58–4.08, *p* < 0.001), obese (AOR = 1.71, 95% CI = 1.05–2.89, *p* = 0.045), diabetes duration > 6 years (AOR = 1.83, 95% CI = 1.20–2.79, *p* = 0.005), TG ≥ 1.7 mmol/l (AOR = 1.81, 95% CI = 1.25–2.78, *p* = 0.019) and LDL-C ≥ 2.6 mmol/l (AOR = 1.55, 95% CI = 1.04–2.28, *p* = 0.039). Moreover, participants who achieved TOC-target indicators significantly had better glycemic control (AOR = 0.69, 95% CI = 0.43–0.89, *p* = 0.034).

**Table 5:** Variables associated with glycemic control by multivariate logistic regression

Covariate	Comparison group	Reduced model			Full model		
		**OR**_**adj**_	**(95% CI)**	***p*-value**	**OR**_**adj**_	**(95% CI)**	***p*-value**
Gender	men vs. women	deleted	-	-	1.03	(0.62, 1.71)	0.906
Age (years)	50–65 vs. < 50	0.51	(0.23, 1.13)	0.095	0.50	(0.22, 1.15)	0.104
	> 65 vs. < 50	0.25	(0.14, 0.55)	< 0.001^***^	0.23	(0.10, 0.52)	< 0.001^***^
BMI (kg/m^2^)	overweight vs. normal	2.54	(1.58, 4.08)	< 0.001^***^	2.65	(1.63, 4.31)	< 0.001^***^
	obese vs. normal	1.71	(1.05, 2.89)	0.045^**^	1.78	(1.03, 3.08)	0.038^**^
Duration of disease (years)	> 6 vs. ≤ 6	1.83	(1.20, 2.79)	0.005^***^	1.91	(1.22, 2.99)	0.005^***^
NC (cm)		deleted	-	-	1.02	(0.96, 1.08)	0.514
WC (cm)	over vs normal	deleted	-	-	1.44	(0.56, 3.69)	0.451
SBP (mmHg)	≥ 140 vs. < 140	deleted	-	-	0.92	(0.41, 2.07)	0.836
DSP (mmHg)	≥ 90 vs. < 90	deleted	-	-	1.99	(0.33, 2.15)	0.456
Diabetes and hypertension	yes vs. no	deleted	-	-	0.92	(0.42, 2.04)	0.845
Underlying diseases	1 underlying disease vs. DM only	deleted	-	-	1.32	(0.49, 3.57)	0.587
	underlying disease more than 2 vs. DM only	deleted	-	-	1.46	(0.49, 4.32)	0.492
DR (> 12 months)	yes vs. no	deleted	-	-	0.69	(0.43, 1.11)	0.127
DN (> 12 months)	yes vs. no	deleted	-	-	0.63	(0.10, 3.93)	0.623
TC (mmol/l)	≥ 5.2 vs. < 5.2	deleted	-	-	1.19	(0.62, 2.31)	0.602
TG (mmol/l)	≥ 1.7 vs. < 1.7	1.81	(1.25, 2.78)	0.019^**^	1.66	(1.09, 2.69)	0.037^**^
HDL-C (mmol/l)	< 1 vs. ≥ 1	1.16	(0.68, 1.97)	0.587	0.91	(0.53, 1.57)	0.738
LDL-C (mmol/l)	≥ 2.6 vs. < 2.6	1.55	(1.04, 2.28)	0.039^**^	1.31	(0.98, 2.22)	0.007
ACEI or ARBs	yes vs. no	deleted	-	-	1.01	(0.60, 1.70)	0.957
Treatment (drug use)	no drug use vs. 1–3	deleted	-	-	0.99	(0.05, 2.52)	0.993
	no drug use vs. 4–5	deleted	-	-	1.05	(0.05, 3.00)	0.974
	no drug use vs. more than 6	deleted	-	-	0.78	(0.04, 1.75)	0.875
**Quality of care indicators**							
TOC-target indicators	yes vs. no	0.69	(0.43, 0.89)	0.034^**^	0.62	(0.21, 0.82)	0.022^**^
POC-process indicators	yes vs. no	deleted	-	-	0.45	(0.39, 0.51)	0.433
**Constant**		1.28	(0.57, 2.90)	0.554	2.16	(0.06, 4.22)	0.680
Hosmer–Lemeshow goodness of fit test		chi-square = 6.74, *p* = 0.257			chi-square = 4.32, *p* = 0.899		
LR test		-239.889			-229.685		
AIC		301.254			431.155		
BIC		453.551			577.003		
Pseudo-R2		0.311			0.261		
Overall Percentage		81.31			77.03%		

^***^ *p*-value < 0.001, ** < 0.05; The association between variables and glycemic control are analyzed by using multivariate logistic regression; *OR* Odd ratio, *CI* Confidence interval, *WC* Waist circumference, *NC* Neck circumference, *SBP* Systolic blood pressure, *DBP* Diastolic blood pressure, *DN* Diabetic nephropathy, *DR* Diabetic retinopathy, *BMI* Body mass index, *TC* Total cholesterol, *TG* Triglyceride, *HDL-C* High-density lipoprotein, *LDL-C* Low-density lipoprotein, *ACEI* Angiotensin-converting enzyme inhibitor, *ARB* Angiotensin receptor blockers, *LR-test* Log likelihood ratio test, *AIC* Akaike’s information criterion, *BIC* Bayesian information criterion

Risk factors significantly associated with DR included obese (I, II) based on BMI, increased DBP and WC (Table 6). Obese participants (AOR = 2.21, 95% CI = 1.00–4.86, *p* = 0.046) and large WC (AOR = 2.23, 95% CI = 0.77–2.31, *p* = 0.021) were strongly correlated with DR. Additionally, participants with higher DBP level were 1.81 times more likely to have DR complication (AOR = 1.81, 95% CI = 1.48–1.96, *p* = 0.049). Therefore, these anthropometric indicators should be closely monitored to prevent and reduce risk of microvascular complications in T2DM patients. Unfortunately, our study did not find any association between DR complication, A1C levels, and the QOC indicators.

**Table 6:** Variables associated with diabetic retinopathy (DR) by multivariate logistic regression

Covariate		Reduced model			Full model		
		**OR**_**adj**_	**(95% CI)**	***p*-value**	**OR**_**adj**_	**(95% CI)**	***p*-value**
Gender	men vs. women	0.74	(0.38, 1.46)	0.388	0.76	(0.35, 1.65)	0.485
Age (years)	60 vs. < 60	deleted	-	-	0.64	(0.31, 1.33)	0.232
BMI (kg/m^2^)	overweight vs. normal	deleted	-	-	0.87	(0.38, 1.99)	0.733
	obese vs. normal	2.21	(1.00, 4.86)	0.046^**^	0.65	(0.25, 1.72)	0.390
Duration of disease (years)	> 4 vs. ≤ 4	1.05	(0.51, 2.17)	0.897	1.23	(0.61, 2.48)	0.557
WC (cm)	over vs. normal	2.23	(0.77, 2.31)	0.021^**^	1.27	(0.54, 2.98)	0.587
NC (cm)		deleted	-	-	0.99	(0.91, 1.06)	0.694
SBP (mmHg)	≥ 140 vs. < 140	deleted	-	-	2.13	(1.02, 4.46)	0.045^**^
DBP (mmHg)	≥ 90 vs. < 90	1.81	(1.48, 1.96)	0.049^**^	0.29	(0.03, 2.45)	0.256
TC (mmol/l)	≥ 5.2 vs. < 5.2	0.47	(0.21, 1.02)	0.051	2.45	(0.82, 7.30)	0.108
TG (mmol/l)	≥ 1.7 vs. < 1.7	0.41	(0.15, 1.10)	0.078	0.53	(0.24, 1.18)	0.122
HDL-C (mmol/l)	< 1 vs. ≥ 1	deleted	-	-	0.65	(0.23, 1.84)	0.420
LDL-C (mmol/l)	≥ 2.6 vs. < 2.6	deleted	-	-	0.49	(0.18, 1.36)	0.171
A1C (mmol/mol)	≥ 53 vs. < 53	deleted	-	-	1.08	(0.53, 2.19)	0.841
Treatment (drug use)	no drug use 1 -3 vs. 4–5	deleted	-	-	1.87	(0.69, 5.07)	0.217
	no drug use 1 -3 vs. more than 6	deleted	-	-	1.75	(0.60, 5.14)	0.306
**Quality of care indicators**							
TOC-target indicators	yes vs. no	deleted	-	-	0.31	(0.11, 1.92)	0.675
POC-process indicators	yes vs. no	deleted	-	-	0.76	(0.44, 1.25)	0.566
**Constant**		0.12	(0.05, 0.30)	0.000	0.14	(0.04, 0.51)	0.003
Hosmer–Lemeshow goodness of fit test		chi-square = 6.14, *p* = 0.5240			chi-square = 4.70, *p* = 0.7896		
-2 Log Likelihood		-126.2933			-125.2933		
AIC		266.5867			270.069		
BIC		293.1164			326.2141		
Pseudo R^2^		0.492			0.543		

^***^ *p*-value < 0.001, ** < 0.05; The association between variables and DR are analyzed by using multivariate logistic regression

*OR* Odd ratio *CI* Confidence interval, *DR* Diabetic retinopathy, *WC* Waist circumference, *NC* Neck circumference, *SBP* Systolic blood pressure, *DBP* Diastolic blood pressure, *BMI* Body mass index, *TC* Total cholesterol, *TG* Triglyceride, *HDL-C* High-density lipoprotein, *LDL-C* Low-density lipoprotein, *A1C* Hemoglobin A1c, *AIC* Akaike’s information criterion, *BIC* Bayesian information criterion

## Discussion

Attainment of good glycemic control and regular screening for comorbidities are essential aspects of T2DM management. These goals help prevent or delay microvascular, macrovascular complications, organ damage, and death [38–40]. Risk factor modification, combined with therapeutic care, improves overall outcomes and well-being. Our study demonstrated that access to critical tests managed by primary care professionals generally reaches acceptable targets (POC and *AllFACE*). The concept of "process of care" in this study was mainly referred to as the receipt of essential diabetes examinations, i.e., the *FACE* indicators. However, from a health services research perspective, the process of care is also a reflection of the physician–patient encounters in the primary care setting which takes into account patients’ healthcare needs and minimize treatment burden. Existing evidence suggests that the sustainability of long-term improvements of glycemic control remains a major challenge in the prevention and control of diabetes and its complications [41, 42]. A recent multi-site evaluation from patients' perspectives demonstrated that an improvement in primary care patients' experiences was significantly associated with reduced treatment burden [43]. Efforts to improve the process of care that takes into account a wider context of service delivery would help in strengthening patients' long-term adherence to diabetic care [44]. Moreover, strengthening the capacity building of primary care physicians and multidisciplinary teams are also important to achieve a more holistic approach towards an optimised delivery of primary care. Interestingly, most patients in this study could access to LDL-C examination and achieve LDL-C target. However, an LDL-C 2.6 mmol/l or lower can be achieved by pharmacological treatment rather than behavioral modification alone in this setting. This is likely because adherence to a healthy lifestyle is challenging especially on maintenance of body weight and healthy dietary consumption [45]. Moreover, optimal glycemic and BP control are more challenging since these two are subject to family and social encouragement and socioeconomic changing, and require multidisciplinary collaboration and patient compliance [46, 47].

A multi-center cross-sectional study in 26,869 Thai patients from 595 hospitals showed that larger hospitals and hospitals having a specialized diabetes clinic outperformed general medical clinics regarding accessibility, effectiveness, and quality of diabetes care. For example, they showed that achievement of essential process of care and quality of care (*AllFACE* and *AllABC)* were reported to be 19.3–29.6% and 8.4–10.5%, respectively [6]. De Berardis et al., showed in a prospective study of 3,437 Italian patients treated by different specialties in 125 diabetes clinics and 103 general practice clinics that care supervised in diabetes clinics related with better clinical outcome goals than general clinics [48]. However, we found that in our getting, achievement of these indicators was 65% and 8%, respectively, especially in the achievement of LDL-C clinical target. This large discrepancy between a proportion of patients who met the POC and TOC may due to the nature of diabetes, financial burden, work and environment related factors. Recent statement from the American College of Lifestyle Medicine suggested that an ability of diabetes control or even diabetes remission is related to its intensity of preventive interventions [11]. Therefore, it is explainable that this result will help us drive the healthcare system that we then need to use our data to implement more intense interventions with patients to improve TOC indicators.

There are possible explanations why diabetes care at these PCUs could outperform larger scale hospital-based clinics in terms of T2DM care. First, primary care physicians and health care professionals in PCUs are familiar with patients with longer-term, doctor-patient relationships. Second, access to medical care in PCUs was limited in the past. Therefore, over the past 4 decades, Thailand has focused on considerable investment and implementation in strengthening its primary health care system, especially at PCUs.

Our findings demonstrated that high BMI, advanced age, duration of diabetes of 6 years or more, TG of 1.7 mmol/l or more, and LDL-C of 2.6 mmol/l or more, are significantly associated with poorer glycemic control. These parameters reflect key characteristics of the metabolic syndrome: poor glycemic control and central obesity [49]. It is well known that time since diagnosis (i.e. disease duration) increases the risk of mortality as well as chronic complications [12, 50–53]. Due to the long asymptomatic stage of diabetes, most diabetic patients fail to achieve a healthy metabolic lifestyle, leading to poor glycemic control and complications. Elevated BMI was associated with progressively higher risk for all T2DM complications, even more so for women than for men [53]. Although our study did not find any association between neck circumference and DM development, its relationship was reported in other studies. In 2021, a prospective multi-center study determined the association between neck circumference and the incidence of cardiovascular events in T2DM patients and were followed up for a 10-year period in Beijing communities. A higher neck circumference was associated with a higher risk of cardiovascular events by 40% in T2DM patients [54]. In 2019, meta-analysis of a cross-sectional design reported that neck circumference was positive correlated with glycemic indicators including fasting plasma glucose, serum fasting insulin level, and A1C [55]. Moreover, neck circumference was also positively correlated with fasting plasma glucose, A1C in patients with hypertension [56]. In 2015, neck circumference was also positive correlated with most factors related to obesity, glucose metabolism, and lipid parameters in both genders [57]. Therefore, neck circumference was easily measured for fat deposit, which might be a predictive risk factor for cardiovascular development and glycemic indicators.

The quality of lipid control was significantly related to glycemic control. It was reported that there was a significant positive correlation between A1C and lipid profile including TC, TG, and LDL-C [58]. Patients with A1C lower than 53.0 mmol/mol had significantly better values of TC, LDL-C, and LDL-C/HDL-C ratio, as compared with ones with A1C > 53.0 mmol/mol [59]. Therefore, the lipid profile could be used as a potential biomarker for predicting glycemic control and the early detection of elevated.

In our study, A1C (≥ 53.0 mmol/mol) was not statistically associated with DR but there were a moderate and a high risk of foot ulcers or wounds. A retrospective cohort study of diabetic patients at the Johns Hopkins Wound Center demonstrated that well-controlled A1C was significantly associated with faster wound-area healing rate [60]. The most common location of wounds was at the lower extremities including legs, ankles and feet. Besides, there may be a relationship between faster wound-healing rate and lower A1C levels [60]. Moreover, the study of T2DM in South Indian using retrospective observational design demonstrated that poor glycemic control was associated with neuropathy, DR and DN [61]. Patients with DN were prone to have higher A1C which was associated with 1.49 times of having DN [12].

Our study has limitations. First, our study was cross-sectional design; therefore, measuring clinical targets, POC, TOC and patients’ characteristic data can be subject to time of assessment which may not represent overall care for all primary care units. Second, at the time of assessment the rate of albuminuria monitoring was low; therefore, we could not determine prevalence of DN and its associated variable risk factors. Third, at PCUs paper-based records were used in parallel with some electronic patient records to support different tasks including anthropometric information and peripheral blood glucose data; therefore, we could not retrieve all data from electronic medical records for the analyses.

In conclusion, in terms of QOC in PCUs, most diabetic patients receive essential diabetes examination but still cannot achieve goals of diabetic control**.** Diabetic retinopathy is a major microvascular complication in an urban population**.** Associated risk factors include diabetes longer than 6 years, obesity with abnormal lipid profiles were significantly at risk for poor glycemic control and diabetic retinopathy. The relationship between glycemic levels and lipid levels can be an early warning indicator for the development of vascular complications in T2DM participants. Therefore, all diabetic patients should be aware of both essential screening and goals of treatment to reduce risk of complications in future**.**

## Data Availability

The datasets used and analyzed during the current study are available from the corresponding author on reasonable request.

## Abbreviations

T2DM: Type 2 diabetic mellitus
NCDs: Non-communicable diseases
NHES: Thai National Health Examination Survey
QOC: Quality of diabetic care
TOC: Clinical targets of care
POC: Processes of care
AOR: Adjust odd ratio
PCUs: Primary care units
DN: Diabetes nephropathy
DR: Diabetes retinopathy
A1C: Glycosylated hemoglobin A1C
ADA: American Diabetes Association
BMI: Body mass index
SBP: Systolic blood pressure
DBP: Diastolic blood pressure
FBG: Fasting blood glucose
TC: Total cholesterol
TG: Triglycerides
HDL-C: High-density lipoprotein cholesterol
LDL-C: Low-density lipoprotein cholesterol
AST: Aspartate transaminase
ALT: Alanine aminotransferase
NPDR: Non-proliferative diabetic retinopathy
PDR: Proliferative diabetic retinopathy
ACR: Albumin-to-creatinine ratio
KDIGO: Kidney Disease Improving Global Outcomes
Cr: Creatinine
eGFR: Estimated glomerular filtration rate
BP: Blood pressure
SEM: Standard error of mean
OR: Odds ratio; CI: confidence interval
WC: Waist circumference
UHC: The universal health care
IOM: The Institute of Medicine
AACN: The American Association of Colleges of Nursing
PCP: Primary care providers
